# Food-Related Compounds That Modulate Expression of Inducible Nitric Oxide Synthase May Act as Its Inhibitors

**DOI:** 10.3390/molecules17078118

**Published:** 2012-07-05

**Authors:** Wilson Maldonado-Rojas, Jesus Olivero-Verbel

**Affiliations:** Environmental and Computational Chemistry Group, University of Cartagena, Cartagena 130015, Colombia; Email: wmaldonador@unicartagena.edu.co

**Keywords:** natural compounds, molecular docking, inflammation, gene expression

## Abstract

Natural compounds commonly found in foods may contribute to protect cells against the deleterious effects of inflammation. These anti-inflammatory properties have been linked to the modulation of transcription factors that control expression of inflammation-related genes, including the inducible nitric oxide synthase (iNOS), rather than a direct inhibitory action on these proteins. In this study, forty two natural dietary compounds, known for their ability to exert an inhibitory effect on the expression of iNOS, have been studied *in silico* as docking ligands on two available 3D structures for this protein (PDB ID: 3E7G and PDB ID: 1NSI). Natural compounds such as silibinin and cyanidin-3-rutinoside and other flavonoids showed the highest theoretical affinities for iNOS. Docking affinity values calculated for several known iNOS inhibitors significatively correlated with their reported half maximal inhibitory concentrations (R = 0.842, *P* < 0.0001), suggesting the computational reliability of the predictions made by our docking simulations. Moreover, docking affinity values for potent iNOS inhibitors are of similar magnitude to those obtained for some studied natural products. Results presented here indicate that, in addition to gene expression modulation of proteins involved in inflammation, some chemicals present in food may be acting by direct binding and possible inhibiting actions on iNOS.

## 1. Introduction

The intake of natural dietary bioactive compounds is associated with low incidence of many diseases. The beneficial biological effects of these chemicals present in fruits and plant-derived-foods may be due to two of their known properties: their affinity for certain proteins and their antioxidant activity. Numerous publications have shown that, in addition to their antioxidant capacity, compounds of plant origin may regulate different signaling pathways in some diseases [1,2,3,4]. Some of this large list of molecules include isothiocyanates, proanthocyanidins, terpenoids, carotenoids, omega-3, and polyunsaturated fatty acids, among others [2,5,6,7,8].

Among most common food-related compounds of particular importance as anti-inflammatory drugs, flavonoids play a pivotal role. These chemicals are a large and diverse group of plant compounds that chemically are derivatives of the benzo-γ-pyrone ring, contain phenolic and pyran groups, conjugation between rings A and B, and differ according to their hydroxyl, methoxy and glycosidic side groups [9]. During metabolism, the free hydroxyl groups may be methylated, sulfated or glucuronidated. In foods, flavonoids are mainly found as 3-*O*-glycosides (anthocyanins) and polymers [10]. These natural compounds have received significant interest due to their known biological properties, in particular the prevention of inflammation [11], cancer [12], and cardiovascular diseases [13]. Several of the anti-inflammatory effects of these molecules have been associated with the inhibition of protein expression of key mediators involved in inflammation processes, such as the inducible nitric oxide synthase (iNOS) [14].

NOSs are a family of eukaryotic enzymes that produce nitric oxide (NO) from L-arginine. In mammals there are three isoforms [15]: the endothelial (eNOS) and neuronal (nNOS) isozymes are constitutively expressed [16], and through the production of low levels of NO, they are involved in the regulation of blood pressure and nerve function, respectively. In contrast, iNOS is produced in response to cytokines or pathogens [17]. NOS contains the heme group in its catalytic site, which is important for its enzymatic activity [18].

Several natural products have been shown to modulate iNOS expression [19]. However, for many of them it remains unclear if the process is the result of an interaction with inflammation-related transcription factors or the enzyme itself. Computational chemistry offers powerful tools to explore these mechanisms by using molecular docking. This method is widely utilized in drug development, and this approach generally assumes a rigid receptor structure to allow the binding of a ligand on putative binding sites on the receptor surface [20,21]. AutoDock Vina has become one of the most frequently used docking software for this purpose. It combines some advantages of knowledge-based potentials and scoring functions, extracting empirical information from the conformational preferences of the receptor-ligand complex, and from experimental affinity measurements. Ligands are ranked based on an energy scoring function and, to speed up the score calculation, a grid-based protein-ligand interaction is employed [22]. In this study, docking methods were used to theoretically evaluate the ability of 42 natural bioactive compounds, known to regulate human iNOS mRNA expression, to bind this protein.

## 2. Results and Discussion

### 2.1. Docking Affinities of Natural Dietary Bioactive Compounds with iNOS

AutoDock Vina-calculated affinity scores obtained for natural bioactive products on examined iNOS structures (PDB ID: 3E7G, PDB ID: 1NSI) are presented in Table 1. Several molecules showed affinity values at least two units greater than the average obtained for all tested compounds, suggesting those may be efficient ligands for iNOS. The best docking results were observed for cyanidin-3-rutinoside (cyanidin-3-rutinoside) (−9.3 kcal/mol for PDB ID: 3E7G, and −9.5 kcal/mol for PDB ID: 1NSI) and silibinin (−9.5 kcal/mol for PDB ID: 3E7G, and −9.2 kcal/mol for PDB ID: 1NSI). However, other molecules such as the anthocyanin cyanidin-3-sambubioside, malvidin-3-arabinoside, malvidin-3-galactoside, petunidin-3-arabinoside, resveratrol and cyanidin, also presented affinity values lower than −8.9 kcal/mol.

**Table 1:** AutoDock Vina-calculated affinities obtained for docking of natural bioactive compounds on iNOS.

Compound	Natural source [References]	3E7G 1NSI Affinity score (Kcal/mol) *^a^*	
Cyanidin-3-rutinoside	Raspberry, cherries [23,24]	−9.3 ± 0.0	−9.5 ± 0.0
Silibinin	Milk thistle [25,26]	−9.5 ± 0.0	−9.2 ± 0.0
Cyanidin-3-sambubioside	Peanut [27]	−9.2 ± 0.0	−8.5 ± 0.0
Malvidin-3-arabinoside	Blueberries [28]	−8.3 ± 0.0	−9.2 ± 0.0
Malvidin-3-galactoside	Berries [29]	−7.9 ± 0.0	−9.1 ± 0.0
Petunidin-3-arabinoside	Bilberry [30]	−8.5 ± 0.0	−9.0 ± 0.0
Resveratrol	Grape skins [31]	−8.9 ± 0.0	−7.5 ± 0.0
Cyanidin	Strawberries [32]	−8.9 ± 0.1	−7.1 ± 0.0
Delphinidin-3-arabinoside	Blueberries [28]	−8.2 ± 0.0	−8.8 ± 0.0
Petunidin-3-glucoside	Blueberries [28]	−8.1 ± 0.0	−8.8 ± 0.0
Peonidin-3-glucoside	Black rice [33]	−8.4 ± 0.0	−8.6 ± 0.0
Malvidin-3-glucoside	Berries [29]	−8.1 ± 0.0	−8.6 ± 0.0
Apigenin	Celery [34]	−8.4 ± 0.0	−7.7 ± 0.0
Carnosol	Rosemary [35]	−8.6 ± 0.0	−7.4 ± 0.0
Delphinidin	Dark berries [36]	−8.6 ± 0.0	−7.0 ± 0.0
Proanthocyanidin	Berries [37]	−8.5 ± 0.0	−8.5 ± 0.0
Epigallocatechin-3-gallate	Green tea [38]	−8.3 ± 0.0	−8.3 ± 0.0
Cyanidin-3-galactoside	Lingonberry [39]	−8.3 ± 0.0	−8.1 ± 0.0
Delphinidin-3-glucoside	Berries [29]	−8.1 ± 0.0	−8.3 ± 0.0
Quercetin	Broccoli [40]	−8.3 ± 0.0	−7.8 ± 0.0
Cyanidin-3-glucoside	Black rice [33]	−8.2 ± 0.0	−8.1 ± 0.0
Pelargonidin-3-glucoside	Strawberries [41]	−8.0 ± 0.0	−8.1 ± 0.0
Curcumin	Curcuma [42]	−8.1 ± 0.1	−7.8 ± 0.1
Kaempferol	Broccoli [40]	−8.1 ± 0.0	−7.7 ± 0.0
5-Hydroxy-3,6,7,8,3',4-hexamethoxyflavone	Citrus peel [43]	−8.1 ± 0.0	−6.5 ± 0.0
All-*trans*-retinoic acid	Carrot [44]	−8.0 ± 0.1	−7.8 ± 0.0
Naringenin	Citrus peel [45]	−8.0 ± 0.0	−7.4 ± 0.0
Pterostilbene	Blueberries [46]	−7.9 ± 0.0	−7.3 ± 0.0
Tangeretin	Citrus peel [47]	−7.5 ± 0.0	−7.0 ± 0.0
Genistein	Soybean [48]	−7.5 ± 0.1	−6.9 ± 0.0
Docosahexaenoic acid	Fish and fish oil [49]	−6.4 ± 0.1	−7.5 ± 0.1
Epicatechin	Green tea [38]	−7.3 ± 0.0	−7.3 ± 0.0
[6]-Shogaol	Ginger [50]	−7.2 ± 0.1	−7.2 ± 0.0
[6]-Gingerol	Ginger [50]	−7.1 ± 0.1	−6.9 ± 0.0
Eicosapentaenoic acid	Fish and fish oil [49]	−6.3 ± 0.1	−7.1 ± 0.1
Phenethylisothiocyanate	Cabbage [51]	−6.1 ± 0.0	−6.1 ± 0.0
Lycopene	Tomato [52]	−6.1 ± 0.2	−4.0 ± 0.2
Benzylisothiocyanate	Cabbage [51]	−6.0 ± 0.0	−5.8 ± 0.0
Menthone	Mentha [53]	−5.8 ± 0.0	−4.7 ± 0.0
Sulforaphane	Cabbage [54]	−4.8 ± 0.0	−4.7 ± 0.0
β-Carotene	Carrot [55]	−4.8 ± 0.1	−0.5 ± 0.4
Lutein	Spinach and eggs [56]	−3.5 ± 0.0	−1.8 ± 0.9
Mean affinity (kcal/mol)		−7.6 ± 0.2	−7.3 ± 0.3
AR-C95791(Inhibitor-iNOS)		−8.4 ± 0.0	−6.9 ± 0.0
L-Arginine (Substrate- iNOS)		−5.9 ± 0.0	−6.4 ± 0.0

^a^ Mean AutoDock Vina affinity value obtained after 10 docking runs per ligand.

Surprisingly, the AutoDock Vina affinity values obtained from docking onto iNOS the ligands AR-C95791 (−8.4 kcal/mol and −6.9 kcal/mol for PDB ID: 3E7G and PDB ID: 1NSI, respectively) and L-arginine (−5.9 kcal/mol and −6.4 kcal/mol for PDB ID: 3E7G and PDB ID: 1NSI, respectively), were similar in magnitude to the average obtained for all 42 tested compounds (PDB ID: 3E7G= −7.6 ± 0.2 kcal/mol and PDB ID: 1NSI= −7.3 ± 0.3 kcal/mol).

### 2.2. iNOS Interacting Residues with Natural Compounds and Search for Allosteric Binding Sites

**Figure 1:**
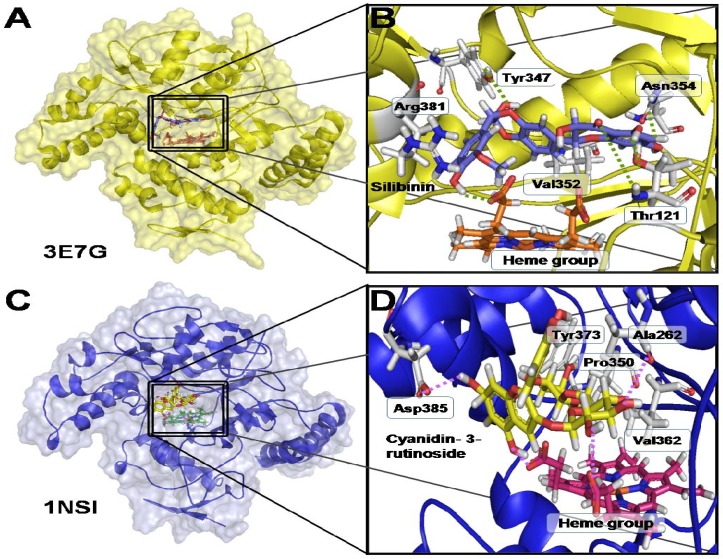
3D view and interacting residues present in the 3E7G/silibinin (**A,B**) and 1NSI/cyanidin-3-rutinoside (**C,D**) complexes.

The interactions observed in the iNOS (3E7G)/silibinin and iNOS (1NSI)/cyanidin-3-rutinoside complexes, as predicted by LigandScout 3.0, are shown in Figure 1. Clearly, the spatial poses acquired by silibinin and cyanidin-3-rutinoside (Figure 1A *vs*. Figure 1C) differ for each protein structure. The most favorable interacting residues with silibinin on 3E7G binding site were Asn354, Thr121, Tyr347 (hydrogen bond donor), Thr121 (hydrogen bond acceptor), Val352 and Arg381 (hydrophobic). Additionally, with respect to the heme group, important constituent of the catalytic site of the iNOS, silibinin showed one interacting hydrogen bond (Figure 1B). In the case of cyanidin-3-rutinoside, interacting residues on 1NSI binding site were Ala262, Tyr373, Asp385 (hydrogen bond donor), Tyr 373 (hydrogen bond acceptor), Pro350 and Val362 (hydrophobic). The 3E7G/silibinin complex also showed interactions with the heme group, in this case three in total (two hydrogen bond acceptor and one hydrophobic interaction) (Figure 1D).

Cyanidin-3-rutinoside and silibinin, molecules with the best absolute affinity values for evaluated iNOS structures, were subsequently submitted to additional docking simulations, using a greater docking box covering the whole protein surface, and a total of 100 runs. This procedure showed that the ligands interacted with the proteins only at the inhibitor/substrate binding site, and no allosteric sites were detected.

The complexes formed between silibinin and cyanidin-3-rutinoside with iNOS structures (PDB ID: 3E7G and PDB ID: 1NSI) involved several types of interactions. This observation implies these natural compounds behave as versatile and efficient ligands for iNOS, as they rely on a diversity of functional groups to completely accommodate into the binding site, besides, presenting interesting interactions with the heme group in the active site for each one of the examined complexes (3E7G-silibinin and 1NSI/cyanidin-3-rutinoside). It is also important to mention that these compounds, which have high affinity scores (lower than −9.2 kcal/mol), interact with the iNOS only at the substrate-inhibitor binding site, suggesting that their action on this enzyme may be related to competitive inhibition with the substrate.

Results presented here have shown that some food-derived molecules with known capacity of regulating iNOS expression, are good ligands for this pro-inflammatory protein. This finding may in part explain some anti-inflammatory effects attributed to the intake of bioactive natural compounds [57,58,59,60,61,62,63]. Cyanidin-3-rutinoside, an anthocyanin present in raspberries and cherries [23,24], and silibinin, also known as silybin, a widely used flavonolignan from milk thistle [25,26], were the compounds with greater *in silico* binding affinities for iNOS. The high binding affinity registered for silibinin (−9.5 kcal/mol for PDB ID: 3E7G), and cyanidin-3-rutinoside, may suggest a possible direct iNOS inhibition, in addition to the experimentally demonstrated down-regulation of the genes [64]. Silibinin has been associated with down-regulation of the iNOS in human lung carcinoma [65]. Moreover, cyanidin-3-rutinoside has been reported to regulate the expression of iNOS and cyclooxygenase-2 (COX-2) in cell-based assays [66,67]. Extracts with high content of pelargonidin-3-glucoside, cyanidins and other anthocyanins, have also been known as inhibitors of iNOS expression in lung carcinoma cells in mice [68]. Blueberry extracts with significant levels of anthocyanins, such as malvidin, petunidin, and peonidin, compounds that are similar to some evaluated here, have been shown to possess the ability to attenuate the expression and activity of iNOS and COX-2 proteins [69]. In the case of iNOS, the inhibitory effect of this extract on enzyme activity has been evaluated, reaching an IC_50_ value of 36 µg/mL [70].

It is important to mention that in addition to the natural compounds present in foods that were examined in this study [19], there are many other naturally occurring chemicals, such as mangiferin, rodgersinol, and withaferin, among others, that have the ability to reduce NO production by attenuating the expression of iNOS [71,72,73].

### 2.3. Docking Validation with Biological Data

It should be pointed out that results from docking analysis only provide theoretical insight about plausible mechanisms involved in the anti-inflammatory properties of these compounds. In order to explore if affinity values calculated by AutoDock Vina may be used as a measure of the likeliness of a particular compound to behave as an iNOS inhibitor, a group of thirty active compounds with confirmed inhibitory activity on iNOS, reported in PubChem BioAssay database [74], were docked to iNOS isoforms (PDB ID: 3E7G and PDB ID: 1NSI), and their affinities calculated by AutoDock Vina [22]. The biological activity of validation compounds comprises a wide range of IC_50_ values, from nanomolar to micromolar concentrations, including values reported for compounds classified as potent inhibitors of iNOS activity [75,76]. Moreover, this activity has been reported to be isoform-specific, as significant differences on enzyme inhibition have been shown when iNOS activity was compared to those elicited by the endothelial nitric oxide synthase (eNOS) and the neuronal nitric oxide synthase (nNOS) [77].

**Figure 2:**
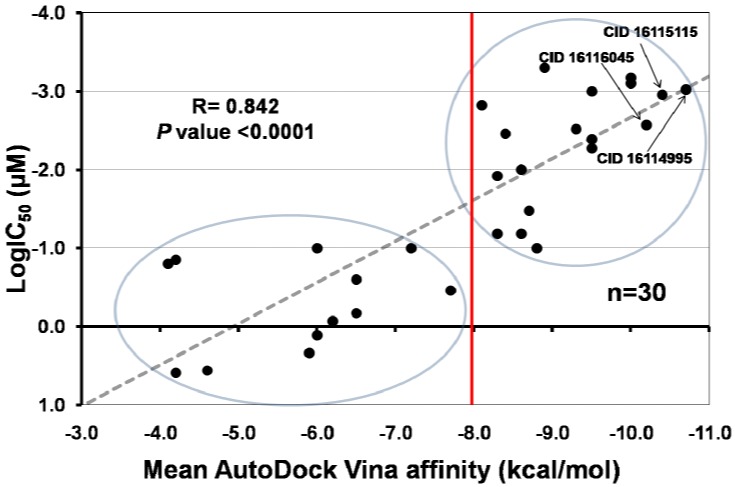
Correlation between the mean affinities calculated by AutoDock Vina in 3E7G and 1NSI for iNOS inhibitors, and their half maximal inhibitory concentration [LogIC_50_]. The regression line (Y = 0.375X + 1.820) was added for illustrative purposes. Circles show molecules with high (upper) and low (lower) biological activity.

The name or PubChem chemical structure identifier (CID), AutoDock Vina affinity value, and biological activity (IC_50_) for reported iNOS inhibitors are presented in Table 2. The relationship between the biological activity (IC_50_) and the mean binding affinity obtained for both iNOS structures are shown in Figure 2. The data indicated the inhibition of iNOS activity follows a linear relationship with the theoretical binding affinity for these compounds. 

**Table 2:** AutoDock Vina-calculated affinities of selected inhibitors for iNOS and theirs half maximal inhibitory concentrations (IC_50_).

iNOS inhibitor	AID/Reference	PDB ID: 3E7G Affinity (kcal/mol)	PDB ID: 1NSI Affinity (kcal/mol)	Affinity mean *^a^*	IC_50_ (µM)	LogIC_50_ (µM)
Pimagedine	AID: 92004	−4.0 ± 0.0	−4.4 ± 0.0	−4.2 ± 0.0	3.9	0.59
AMT	[78]	−4.7 ± 0.1	−4.4 ± 0.1	−4.6 ± 0.1	3.6	0.56
N(G)-iminoethylornithine	AID: 92181	−5.5 ± 0.0	−6.2 ± 0.0	−5.9 ± 0.1	2.2	0.34
L-NIL	AID: 92009	−5.8 ± 0.1	−6.3 ± 0.1	−6.0 ± 0.1	1.3	0.11
Targinine	AID: 92143	−5.8 ± 0.1	−6.7 ± 0.0	−6.2 ± 0.1	0.86	−0.07
Nitroarginine	AID: 92143	−6.1 ± 0.1	−6.9 ± 0.0	−6.5 ± 0.1	0.67	−0.17
AR-C95791	AID: 92009	−8.4 ± 0.0	−6.9 ± 0.0	−7.7 ± 0.2	0.35	−0.46
CID10398018	AID: 92144	−6.4 ± 0.0	6.6 ± 0.1	−6.5 ± 0.0	0.25	−0.60
Etiron	AID: 92011	−4.1 ± 0.1	−4.1 ± 0.0	−4.1 ± 0.0	0.16	−0.80
CID 10011896	AID: 92011	−4.2 ± 0.1	−4.3 ± 0.0	−4.2 ± 0.0	0.14	−0.85
CID 3863	AID: 92004	−5.9 ± 0.0	−6.2 ± 0.1	−6.0 ± 0.0	0.1	−1.00
CID 16116298	AID: 280474	−7.6 ± 0.0	−6.7 ± 0.0	−7.2 ± 0.1	0.1	−1.00
CID 16116293	AID: 280474	−8.4 ± 0.0	−9.2 ± 0.0	−8.8 ± 0.1	0.1	−1.00
CID 16115471	AID: 280474	−8.1 ± 0.1	−8.6 ± 0.0	−8.3 ± 0.1	0.066	−1.18
CID 16115345	AID: 280474	−8.1 ± 0.0	−9.0 ± 0.0	−8.6 ± 0.1	0.066	−1.18
CID 44420709	AID: 280474	−8.7 ± 0.0	−8.7 ± 0.0	−8.7 ± 0.0	0.033	−1.48
CID 16116564	AID: 280474	−8.1 ± 0.0	−8.5 ± 0.0	−8.3 ± 0.0	0.012	−1.92
CID 16115897	AID: 280474	−8.3 ± 0.0	−8.9 ± 0.0	−8.6 ± 0.1	0.01	−2.00
CID 16115611	AID: 280474	−9.8 ± 0.0	−9.1 ± 0.0	−9.5 ± 0.1	0.0054	−2.27
CID 16115606	AID: 280474	−8.6 ± 0.0	−9.6 ± 0.0	−9.5 ± 0.1	0.0041	−2.39
CID 16115472	AID: 280474	−8.1 ± 0.0	−8.8 ± 0.0	−8.4 ± 0.1	0.0035	−2.46
CID 16115342	AID: 280474	−9.5 ± 0.0	−9.1 ± 0.0	−9.3 ± 0.0	0.003	−2.52
CID 16114996	AID: 280474	−9.9 ± 0.0	−10.5 ± 0.0	−10.2 ± 0.0	0.0027	−2.57
CID 16115233	AID: 280474	−7.7 ± 0.0	−8.4 ± 0.0	−8.1 ± 0.1	0.0015	−2.82
CID 16115115	AID: 280474	−10.4 ± 0.1	−10.4 ± 0.0	−10.4 ± 0.0	0.0011	−2.96
CID 16114992	AID: 280474	−9.2 ± 0.0	−9.9 ± 0.0	−9.5 ± 0.1	0.001	−3.00
CID 16114995	AID: 280474	−10.2 ± 0.0	−11.1 ± 0.0	−10.7 ± 0.1	0.00096	−3.02
CID 16116046	AID: 280474	−9.9 ± 0.1	−10.1 ± 0.0	−10.0 ± 0.0	0.0008	−3.10
CID 16116045	AID: 280474	−9.8 ± 0.0	−10.3 ± 0.0	−10.0 ± 0.1	0.00067	−3.17
CID 16115896	AID: 280474	−8.7 ± 0.0	−9.1 ± 0.0	−8.9 ± 0.1	0.0005	−3.30

*^a^* Average affinity between the scores obtained for two iNOS structures (PDB ID: 3E7G and PDB ID: 1NSI), AID: Assay ID (PubChem Bioassay), CID: Compound ID (PubChem Compound), IC_50_: Half maximal inhibitory concentration.

The relationship observed between biological activity (logIC_50_) and *in silico* binding affinity values for known iNOS inhibitors is mostly linear in nature (Figure 2), and our results (R = 0.842, *P* < 0.0001) are much better than those reported for similar studies [79]. The data revealed ligands can be divided in two groups based on their affinity scores. Molecules with affinity scores lower than −8.0 kcal/mol are likely to have IC_50_ values equal or lower than 0.1 µM. In contrast, those with values higher than −8.0 kcal/mol will have less chance to inhibit iNOS. Moreover, those natural compounds with affinity scores lower than −9.0 kcal/mol, such as CID 16116045, CID 16115115, and CID 16114995 are good candidates to have IC_50_ values in the sub nanomolar range. 

Interestingly, two natural compounds evaluated here (silibinin and cyanidin-3-rutinoside) presented affinity scores lower than −9.2 kcal/mol for both iNOS. These values are even better than those obtained for well known inhibitors such as pimagedine, AMT, L-NIL, nitroarginine, targinine, and etiron [74,78]. These results could help explaining some of their benefic effects on human health, not only by their known modulation of transcription factors [19], but also by their behavior as iNOS inhibitors. In general, flavonoids and anthocyanins present good theoretically capacity to bind and inhibit iNOS. Although the anti-inflammatory properties of natural compounds may occur through multiple mechanisms, the exploration of a direct action at the protein level by using docking simulations provides insights regarding their pharmacological benefits. 

## 3. Experimental

### 3.1. Protein Structures and Modeling of Ligands

**Figure 3:**
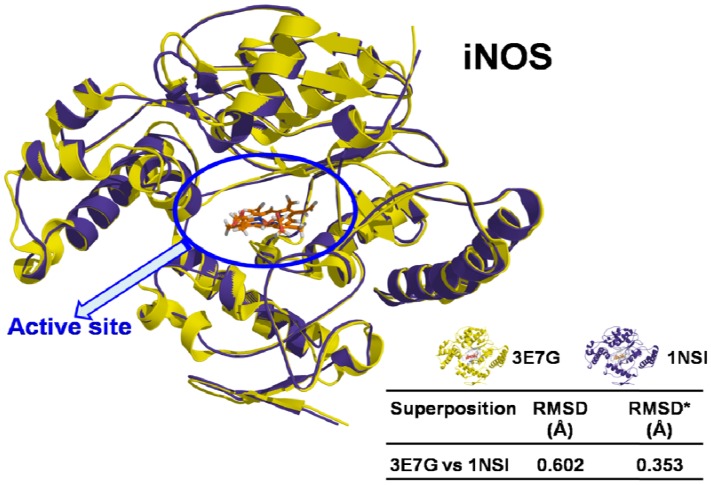
Superposition of iNOS structures (3E7G and 1NSI), showing sequence identity and RMSD values. * RMSD for the binding site.

3D structures of two human iNOS (PDB ID: 3E7G and PDB ID: 1NSI) were downloaded from Protein Data Bank (PDB) [80], prepared and aligned with Sybyl 8.1.1 program [81]. Both models have the same 3D coordinates (sequence identity = 100% and RMSD < 0.459 Å) (Figure 3), and minimal differences can be attributed to the resolution quality for each one (PDB ID: 3E7G = 2.20 Å and PDB ID: 1NSI = 2.55 Å).

**Figure 4:**
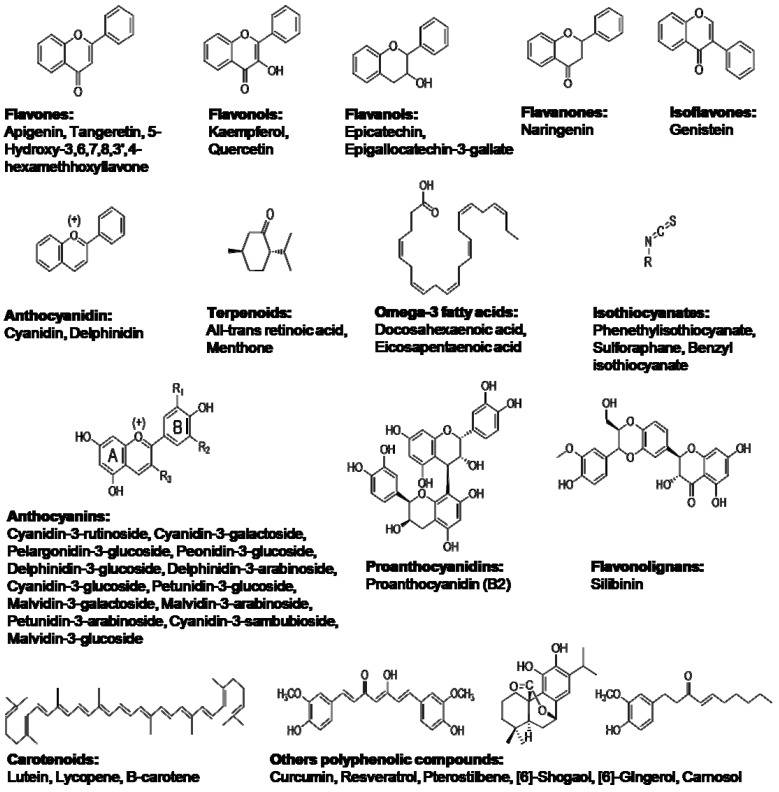
Chemical groups for food-related natural compounds used to perform docking studies on iNOS.

Forty-two anti-inflammatory natural compounds, present in fruits and plant-derived-foods, belonging to different chemical groups (Figure 4), were chosen as protein ligands to perform this study, as they have been reported to modulate expression of genes related to inflammation [19,82]. These compounds comprise distinct chemical families, such as anthocyanins, flavolignans, flavones, and organosulfur compounds, among others. They are considered of special relevance to food and pharmaceutical industry because of their potential health-promoting effects, and favourable organoleptic properties. The geometries of these bioactive compounds were optimized using DFT at the B3LYP/6-31G level [83], and calculations were carried out with Gaussian 03 package program [84]. Open Babel was used to transform geometries to Mol2 format for their subsequent processing [85].

### 3.2. Protein-Ligand Docking Simulations

Molecular docking was utilized to evaluate the feasibility of some food-related natural compounds to form complexes with iNOS. MGL tools 1.5.0 [86] was employed to prepare protein structures for molecular docking, and protein-ligand docking calculations were performed with AutoDock Vina 1.0 program [22], using, as the docking box, the cavity filled by the ligand in the PDB structure (PDB ID: 3E7G and PDB ID: 1NSI). The docking site for the ligands on iNOS structure (PDB ID: 3E7G) was defined by establishing a cube with the dimensions 24 × 24 × 24 Å, covering the ligand binding site with a grid point spacing of 1.0 Å, and center grid boxes of 56.030, 20.374 and 79.669 in X, Y and Z dimensions, respectively. The binding site for the other iNOS structure (PDB ID: 1NSI) was defined similarly to PDB ID: 3E7G, except for the location of the center grid boxes, that in this case were 11.664, 63.188, and 15.995 in X, Y and Z dimensions, respectively. All calculations with AutoDock Vina included 20 number modes, an energy range of 1.5, and exhaustiveness equal to 20. Ten docking runs were executed per each ligand, saving the best obtained pose. The average affinity for best poses was computed as the affinity value for a given complex. For comparison purposes, these calculations were performed for the iNOS inhibitor AR-C95791, as well as for the natural substrate L-arginine. These molecules were extracted from their 3D complex structures, as these were the ligands bound to PDB ID: 3E7G and PDB ID: 1NSI, respectively.

### 3.3. Residues Interacting with the Natural Bioactive Compounds on iNOS Binding Site and Searching of Alternative Allosteric Binding Sites

The identification of protein residues (PDB ID: 3E7G and PDB ID: 1NSI) that interact on the binding site with those natural bioactive compounds that produced the best AutoDock Vina affinity values, was accomplished using LigandScout 3.0 [87], a package that utilizes pharmacophores to establish ligand-aminoacid interactions on the target binding site. Residue-ligand interactions were visualized with PyMol program [88].

In addition, ligands for which docking complexes produced affinity values lower than −9.2 kcal/mol were subjected to docking simulations (n = 100) over the whole protein surface, in order to identify possible allosteric binding sites. This was done by performing a docking simulation as follows: the binding site for the ligand on iNOS (PDB ID: 3E7G, chain A) was defined by forming a cube with the dimensions 66 × 66 × 80 Å, engulfing the whole protein structure, using a grid point spacing of 1.0 Å and center grid boxes of 60.398, 20.038 and 83.489, in X, Y and Z dimensions, respectively. The same approach was used for iNOS structure (PDB ID: 1NSI, chain A), but the dimensions of the cube were 66 × 80 × 66 Å, and the center grid boxes were 12.344, 58.446 and 15.573 in X, Y and Z dimensions, respectively. All other docking parameters were the same as previously described. 

### 3.4. Docking Validation with Biological Data for iNOS Inhibitors

To validate the docking procedure, the 3D structures and the biological data of thirty iNOS inhibitors were obtained from PubChem chemical library [74] and literature [78]. Docking procedures were performed with AutoDock Vina [22], following the same protocols described for natural products. The biological data consisted of the half maximal inhibitory concentration (IC_50_) for iNOS activity, as reported for different compounds in PubChem BioAssay [74]. Detailed information about the synthesis and purification of these iNOS inhibitors has been reported in the literature [77,89]. These assays were conducted on A172 cells, and the activity of iNOS was induced by gamma interferon, tumor necrosis factor alpha, and interleukin 1-beta. Concomitant with cytokine addition, appropriate concentrations of compounds were included. The IC_50_ values were calculated from log-logit analysis of the data [77]. 

Correlation analysis [90] was used to establish relationships between AutoDock Vina-derived affinities of inhibitors on the two tested iNOS (average values) and experimental biological data (LogIC_50_). Statistical analysis was performed using Graph Instat Software [91]. 

## 4. Conclusions

Docking analysis data suggested that it is plausible that in addition to their role mediating transcription regulation of inflammation-related genes, some food-related anthocyanins, such as silibinin and cyanidin-3-rutinoside, may exert direct inhibitory action on iNOS. Future research will focus on experimental evaluation of these results, using the iNOS model to design new derivatives of these natural bioactive compounds.
